# Bet v 1 Allergen Is the Main Driver of Disease in Patients with Pollen Allergy in Belgium

**DOI:** 10.3390/cells15201907

**Published:** 2026-10-20

**Authors:** Françoise Pirson, Esmeralda Fraille Lopez, Cloé Hupin, Carine Sohy, Bertrand Bearzatto, Marylène Lecocq, Charles Pilette

**Affiliations:** 1 Department of Pulmonary Medicine, Cliniques Universitaires Saint-Luc, 1200 Brussels, Belgium; charles.pilette@saintluc.uclouvain.be; 2 Allergy Center Saint-Luc, Catholic University of Louvain (UCLouvain), 1200 Brussels, Belgium; cloehupin@hotmail.com (C.H.); bertrand.bearzatto@uclouvain.be (B.B.); marylene.lecocq@uclouvain.be (M.L.); 3 Lung, Nose and Skin Research Unit (LUNS), Institute of Experimental and Clinical Research (IREC), Catholic University of Louvain (UCLouvain), 1200 Brussels, Belgium; carine.sohy@chuuclnamur.uclouvain.be; 4 Faculty of Medicine, Campus of Woluwé-Saint-Lambert, Catholic University of Louvain (UCLouvain), 1200 Brussels, Belgium; esmeralda.fraille.lopez@gmail.com; 5 Dispens′s Soins, 6200 Châtelet, Belgium; 6 Department of Otorhinolaryngology, Cliniques Universitaires Saint-Luc, 1200 Brussels, Belgium; 7 Department of Otgorhinolaryngology, Hôpital Delta, 1160 Brussels, Belgium; 8 Department of Pulmonary Medicine, University Hospital Center UCL Namur, Godinne Site, 5530 Yvoir, Belgium; 9 Allergy Center Godinne, University Hospital Center UCL Namur, Godinne Site, 5330 Yvoir, Belgium; 10 Experimental and Clinical Research Institute (IREC), Center for Applied Molecular Technologies, Catholic University of Louvain (UCLouvain), 1200 Brussels, Belgium

**Keywords:** allergen components, Bet v 1, IgE sensitization, plant-related food allergy, pollen-allergic patients, profilin

## Abstract

**Highlights:**

**What are the main findings?**

- Bet v 1-sensitization governs the prevalence and severity of respiratory and pollen-related food allergy symptoms in birch and mixed pollen-allergic patients, with particularly more severe plant-related food allergy in birch pollen-allergic patients with asthma. It influences the prevalence of pollen-related food allergy in grass pollen-allergic patients to a lower extent and independently of symptom severity.
- IgE co-sensitization to Bet v 1 and to at least two profilins tends to increase the prevalence of plant-related food allergy, together with the duration of rhinitis and food allergy compared to non-co-sensitized grass pollen-allergic patients, although there is no correlation with the severity of food and respiratory symptoms.

**What are the implications of the main findings?**

- It is important to distinguish between pollen sensitization and pollen allergy.
- Our study did not prove that profilin IgE sensitization is associated with more severe symptoms of respiratory and pollen-related food allergy in grass pollen-allergic patients.

**Abstract:**

Birch pollen-sensitized patients co-sensitized to Bet v 1 and Bet v 2 allergens present more frequent and severe respiratory or plant-related food allergy symptoms (PFASs). This study analyzed the relevance of Bet v 1 and profilin sensitization in the clinical phenotype of pollen-allergic patients in Belgium. Ten patients allergic to birch pollen (BPA), 31 to grass pollen (GPA) and 33 to both pollens (MPA), with rhino-conjunctivitis with/without asthma (ARRC), were evaluated by means of questionnaire, skin-prick tests and specific IgE assays. Half of GPA were co-sensitized to birch pollen, and no BPA were sensitized to profilin. The prevalence of asthma and pollen-related food allergy syndrome (PFAS) was higher in BPA than in MPA or GPA, with a higher severity of PFAS in asthmatic BPA. GPA with PFAS were more frequently sensitized to Bet v 1, with higher levels of rBet v 1-IgE. The prevalence of PFAS, together with the duration of rhinitis and PFAS tended to increase in profilin-polysensitized vs. mono- and un-sensitized GPA, regardless of Bet v 1-IgE and the severity of symptoms.

## 1. Introduction

In Europe, the *Poaceae* and *Betulaceae* families are the most common causes of allergic rhino-conjunctivitis in Central and Northern Europe, including in Belgium [1,2,3,4,5,6,7]. Climatic and geographical factors influence the respiratory phenotype of pollen allergy in relation to the IgE sensitization to pollen allergens and allergenic components [1,8,9,10,11]. These are also linked to symptoms of pollen-related food allergy syndrome (PFAS), frequently observed in pollen-allergic patients [12,13,14,15,16,17]. Belgium is located midway along a North–South axis in Europe, with a warming temperate maritime climate.

The PR-10 protein Bet v 1 is largely responsible for PFAS in birch pollen-allergic patients (BPA), while cross-reactive profilins (Bet v 2, Phl p 12) are more associated with PFAS in grass pollen-allergic patients (GPA), particularly in Southern Europe [4,15,17,18,19]. In a recent study in Germany, patients sensitized to birch pollen (BP), with co-sensitization to Bet v 1 and Bet v 2, were more often affected by oral allergy syndrome (OAS) and intestinal symptoms (but no anaphylaxis) than those without profilin sensitization [20]. They also suffered more frequently and severely from rhinitis, with a tendency to develop asthma. Other studies performed in Southern Europe showed that sensitization to profilins was a marker for the severity of pollen-allergic asthma and rhino-conjunctivitis, with more severe food allergy during an intense grass pollen (GP) season [15,21,22].

Based on the type of clinical allergy (and not only sensitization), our study focused on two objectives, namely (a) to analyze the respiratory phenotype of patients allergic to BP and/or GP in relation to their IgE sensitization profile (allergens and components), and (b) to correlate the symptoms of PFAS with the IgE sensitization profile, particularly taking into account the role of profilins. The study was conducted approximatively 10 years after the first respiratory and food allergy phenotype modifications observed in Europe at the end of the 20th century [2,4,8,12,13]. This timeframe presupposed that the general population had had time to be exposed to the first climatic changes.

## 2. Materials and Methods

### 2.1. Study Design and Selection of Participants

Between 10 May 2010 and 26 January 2011, patients were consecutively recruited from outpatient clinics (chest and ENT departments) at two Belgian academic centers. Inclusion criteria were: well-defined rhinitis and/or conjunctivitis with/without asthma (AARC), consistent with confirmed IgE sensitization to *Betulaceae* and/or *Poaceae* pollens, and present for more than one year over the period from March to July. Patients described the chronology of the symptoms in relation to personal and social events, allowing their timing and reproducibility to be accurately determined. The timing of symptoms and daily pollen concentrations [provided each year by the Mycology and Aerobioloy Department (https://airallergy.sciensano.be/ accessed on 8 March 2013) of the Belgian Scientific Institute of Public Health (WIV-ISP)], confirmed by the skin and laboratory tests, helped define the type of pollinosis. Age, gender, ethnicity and severe symptoms were not exclusion criteria. All patients were administered a standardized questionnaire, skin-prick tests (SPTs) for aeroallergens and skin prick-prick tests (SPPTs) to 7 preselected fresh foods (and any additional clinically relevant foods). The severity of AARC was evaluated based on the ARIA and GINA classifications [23,24]. A blood sample was collected for IgE assays. All participants (or the legal guardians for children) gave their written informed consent before the start of the study.

### 2.2. Skin Testing

Sensitization to aeroallergens was assessed by SPT performed on the forearms with the Stallerpoint^®^ and the commercial extracts (Stallergènes^®^, Antony, France) *Dermatophagoides pteronyssinus*, *Dermatophagoides farinae*, cat and dog dander, pollens of alder, birch, hazel, ash, and mugwort, 5 GP mix, latex, *Alternaria* and *Cladosporium* sp., including glycerinated serum and 9% codeine phosphate as negative and positive controls, respectively. SPPTs were performed in all patients for hazelnut, peanut, Golden Delicious apple, peach, green kiwi, Alpro^®^ soy milk, and raw carrot; other foods were skin tested as clinically relevant. The tests were performed at distance of any antihistamine intake (>10 days), dermal corticosteroid or calcineurin inhibitor use (>1 month) with a reading after 20 min. A positive response was defined by a mean wheal diameter of ≥3 mm larger than the mean diameter of the negative control (and a positive reaction to codeine phosphate).

### 2.3. Serum IgE Assays

The patient’s serum was collected at the time of enrollment, frozen at −20 °C, and the samples were analyzed simultaneously for all patients at the end of 2011. The determination of total (_tot_IgE) and specific (_s_IgE) IgE was performed by ImmunoCAP^TM^ assay (Thermo Fisher Scientific-Phadia AB, Uppsala, Sweden) for the following allergens: *Phleum pratense* (Phl p) or Timothy grass pollen (TGP), *Betula verrucosa* or birch pollen (BP), allergen components of Phl p: rPhl p 1, rPhl p 2, nPhl p 4, rPhl p 5b, rPhl p 6, rPhl p 7, rPhl p 11, rPhl p 12, components of BP rBet v 1, rBet v 2, rBet v 4, rBet v 6, and MUXF3 (for CCD or cross-reactive carbohydrate determinant), and expressed in kU_A_/L with a cut-off at 0.35 kU_A_/L). These components corresponded to the major and minor allergens of the pollens studied, available for IgE assay and referenced in the 2011 product catalog of Thermo Fisher Scientific-Phadia AB. In addition, ImmunoCAP^TM^ ISAC103 (Thermo Fisher Scientific-Phadia AB, Uppsala, Sweden, catalog 2011, Ref.81-10005-01) was used as a semi-quantitative multiplex testing method for 103 allergen components, expressed in ISAC standardized units (ISU; positive > 0.3 ISU).

### 2.4. Statistical Analysis

The non-parametric Mann–Whitney U and Kruskal–Wallis tests were used for the comparison of median values between independent groups, and the parametric Student’s *t* test for the comparison of 2 independent serial data with normal distribution. Comparison of the distribution of qualitative criteria in 2 or more populations was performed by the Chi-squared test (Chi^2^) or Fisher’s exact test (for small groups). The effect size on the mean value ± SD of 2 groups was evaluated using the Cohen’s d test and Hedges’ g test (for small populations). The Cramer’s V (or adjusted Cramer’s V) test was used on categorial data in 2 (or 3) populations. The evaluation of the effect size measured by a Kruskal–Wallis rank sum test on 3 populations was expressed by the epsilon-squared (ε^2^). The adjusted comparisons were performed using multiple linear regression (continuous variables) and logistic regression for categorial outcomes (odds ratio calculation (OR) with [95% CI] and *p*-value). The significance limit was defined as *p* = 0.05. Correlations were established between profilin IgE values with Spearman’s rho rank test. Comparison of correlations was performed using Fisher’s transformation, followed by the Z-test. All statistical analyses were performed using “R++, l’essentiel” for Windows, version 1.7.04 to 1.9.07 (Toulouse, France) and Microsoft Excel^®^ 2021, version 2608/MSO, Microsoft Corporation, (Redmond, WA, USA).

GenAI was not used for this publication.

## 3. Results

### 3.1. Demographic Data

During the limited inclusion period, 87 patients signed an informed consent form; 13 were excluded due to inconsistencies between the symptom period and the nature of pollen IgE sensitization. Seventy-four patients with well-defined respiratory symptoms were included and divided into three groups according to the following criteria:

Birch pollen-allergic patients (BPA), with allergic airway disease from March to April, and positive SPT for ≥two of the three *Betulaceae* pollen extracts, including BP (*n* = 10).

Grass pollen-allergic patients (GPA), with symptoms from May to July, and a positive SPT for the five GP extracts (*n* = 31).

Mixed pollen-allergic patients (MPA), with symptoms from March to July, and a positive SPT for *Betulaceae* pollens (≥two extracts including BP) and five GP extracts (*n* = 33).

The demographic data of the population with pollen-induced AARC and patient clinical characteristics are shown in Table 1, and the sub-populations with AARC and PFAS, in Table 2. Gender distribution was similar in the three groups. Although GPA patients were significantly younger than patients in the other two groups (median effect size measured by the Krukal–Wallis *p* = 0.004; ε^2^ = 0.09, [0.02, 1.00]), median age was similar for the three groups of patients with asthma, PFAS, or both. Seasonal allergic rhino-conjunctivitis was the dominant respiratory disease observed among the three groups, mostly moderate to severe persistent rhinitis. The prevalence of asthma (Chi^2^ *p* = 0.019; Adjusted Cramer’s v = 0.42, [0.17, 1.00]) and PFAS (Adjusted Cramer’s v = 0.28, [0.0, 1.00]) was significantly higher in BP and MPA than in GPA.

### 3.2. Skin-Prick Sensitization Profile

Based on the SPT for aeroallergens, monosensitized patients represented one out of 10 BPAs and three out of 31 GPAs. In the BPA and GPA groups, five out of 10 and 16 out of 31 were respectively co-sensitized to the other pollen, but without related symptoms (Figure 1).

### 3.3. Serum IgE Sensitization Profile

All BPA and GPA patients had positive _s_IgE for BP and TGP respectively, but 30% of BPA were sensitized to TGP and 54.8% of GPA had positive BP-IgE. In BPA, rBet v 1 was the only major allergen (100%), 40% were sensitized to rPhl p 1. In GPA, rPhl p 1, nPhl p 4, rPhl p 2, rPhl p 5b, and rPhl p 6 were major allergens and 48.4% of the patients were co-sensitized to rBet v 1. In MPA, rPhl p 1, rBet v 1, nPhl p 4, rPhl p 5b, and rPhl p 2 were major allergens while the prevalence of sensitization to rPhl p 6 was ~50% (Figure 2a–c).

Median BP-IgE and rBet v 1-IgE levels were significantly higher in BPA vs. GPA (*p* < 0.0005) without difference between the BPA and MPA groups. There was no sensitization to profilins (rBet v 2, rPhl p 12), or to CCD in BPA, whereas non-significant sensitization to isoflavone reductase (rBet v 6) was observed in 10% of BPA with low rBet v6-IgE levels. In GPA, _s_IgE levels for TGP, rPhl p 1, nPhl p 4, and rPhl p 6 were significantly higher than those of MPA and BPA, and those for rPhl p 2 and rPhl p 5b were higher than those of BPA, but similar to those of MPA. In GPA and MPA, the prevalence of sensitization to profilins based on the ImmunoCAP^TM^ assay (rBet v 2, rPhl p 12) was ~24%, ~5% to polcalcins (rBet v 4, rPhl p 7), and 12 to 19% for CCD (in MPA and GPA, respectively) (Table 3 and Table S1).

### 3.4. Food Allergy in Pollen-Allergic Patients

Pollen-allergic patients with PFAS, accounting for 43 out of 74 patients, were significantly older (Cohen’s d of −0.98, 95% CI [−1.5, 0.45], large effect size) with later onset of respiratory disease compared to patients without PFAS (*p* < 0.005 with a Cohen’s d −0.77, [−1.25, 0.29], medium to large effect size) (Table 4). The presence of active asthma was significantly higher in the population with PFAS (OR: 3.2, 95 CI [1.2, 8.3], *p* < 0.05]). The logistic regression analysis revealed the significant association between the development of PFAS with the presence of asthma (OR: 3.2, [1.1, 9.3], *p* < 0.05) and the age at inclusion (OR 1.1, [1, 1.1], *p* < 0.005). The presence of PFAS was independent of sex.

In BPA, the most frequent allergenic foods were raw apple, hazelnut, and cherry, compared with hazelnut, *Rosaceae*, and kiwi in GPA (Table S2). Oral allergy syndrome was the dominant symptom of PFAS in the three groups (Table 2). In the groups with PFAS and subgroups with PFAS and asthma, respiratory symptoms (rhino-conjunctivitis, asthma, wheezing, dyspnea, and laryngeal edema) associated with food ingestion or peeling were significantly more prevalent among BPA (≥50%, *p* < 0.05). These were reported with apple, potato, carrot, soy milk and kiwi. All BPA and three of the four MPA with these symptoms were also asthmatic. More severe digestive symptoms (mainly epigastric discomfort, diarrhea, vomiting, and oropharyngeal edema) were rare, observed in only one asthmatic BPA and three asthmatic MPA, irrespective of the IgE sensitization to profilins. There were no reports of anaphylaxis for any of these patients.

Pollen-allergic patients with PFAS had a significantly higher prevalence of positive BP-, rBet v 1-, rBet v 6-IgE (respective OR with [95% CI] and *p*-value—OR 34.6, [4.21–284], *p* = 0.001; OR 14.2 [3.62–55.9], *p* = 0.0001; and OR 10.9 [0.59–201.5], *p* = 0.10)—and significantly higher BP- and rBet v 1-, and lower rPhl p 2-IgE mean levels than patients without PFAS. A large effect size is observed for BP- and rBet v 1-IgE levels with Cohen’s d values of 0.99 and 0.91, respectively, and a medium effect size is observed for rPhl p2 (d, 0.60) (Table 4, Figure 3a,b). The prevalence of IgE sensitization to the thaumatin-like protein nAct d2, lipid transfer proteins and different classes of storage proteins—7S globulin, 11S globulin and 2S albumin—was not significantly different between patients with or without PFAS, and the levels remained low and not significantly different (Table S3).

The BPA, GPA, and MPA groups were analyzed (Figure 4a–d) according to the presence or absence of PFAS, with comparison performed between subgroups. A larger prevalence of sensitization to rBet v 1 (OR 6.5, [1.09–38.6], *p* < 0.05), a higher level of BP and rBet v 1-IgE (Hedges’ g 0.61 and 0.60 respectively), and a discreetly but significantly higher level of rPhl p 12- and rBet v 2-IgE (Hedges’ g of 0.64 and 0.62, respectively) were observed in the GPA with PFAS compared to those without PFAS. The BP-IgE and rBet v 1-IgE levels were higher in MPA with PFAS vs. without PFAS (Hedges’s g of 0.80 and 0.68 respectively). In GPA, nine out of 10 patients with PFAS were sensitized to BP, including eight sensitized to rBet v 1 and four co-sensitized to profilins. GPA without PFAS had higher rPhl p 2-IgE levels than GPA with PFAS (Hedges’s g of 0.62).

### 3.5. Influence of the IgE Sensitization to Profilins With or Without Co-Sensitization to Bet v 1

In pollen-allergic patients sensitized to rBet v 1 without any sensitization to profilin (*n* = 43), the prevalence of asthma and the severity of rhinitis were significantly higher in BPA and MPA compared with GPA (Adjusted Cramer’s v for asthma: 0.37, [0.0–1] corresponding to a medium effect size). BPA and MPA had also higher levels of rBet v 1-IgE (Table S4). Although the prevalence of PFAS was statistically similar between the three groups, the respiratory symptoms due to plant food ingestion or peeling were more frequent in BPA (Adjusted Cramer’s v: 0.36,95 [0.0–1.0], medium effect size). The phenotype was similar between GPA and MPA co-sensitized to rBet v 1, rPhl p 12, and/or rBet v 2 (*n* = 13), In GPA sensitized to rBet v 1, there was no documented evidence that the co-sensitization to at least one profilin was associated with a higher risk of severe rhinitis, severe asthma, or PFAS compared with GPA not co-sensitized to profilin(s). The relatively high prevalence (80%) of PFAS in GPA co-sensitized to profilin(s) was also associated with a high rBet v 1-IgE level equivalent to the levels in MPA.

**Figure 4:**
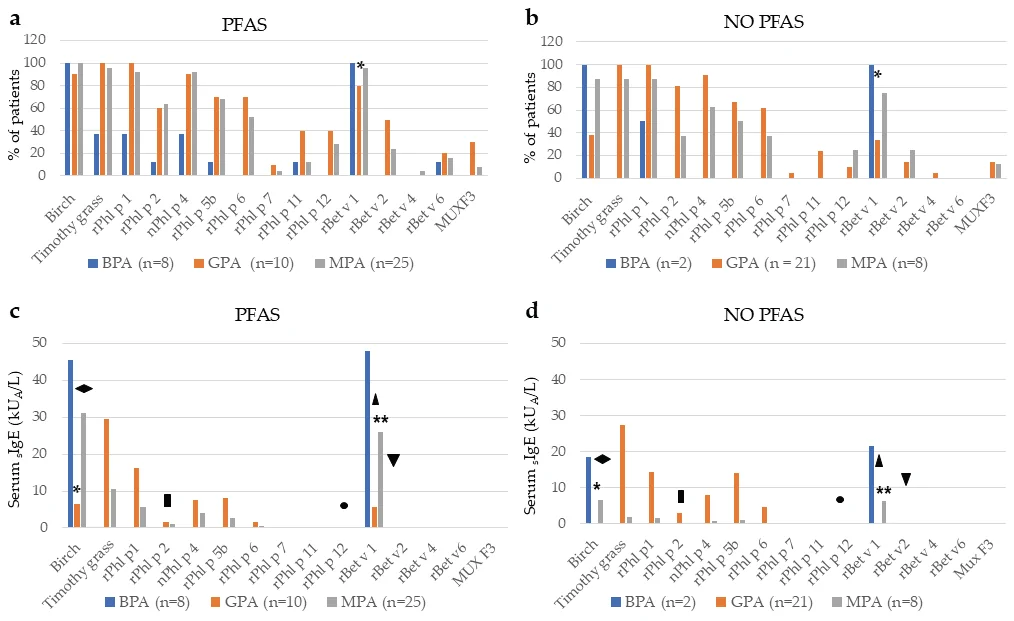
IgE sensitization to pollens and allergen components in the 3 populations of pollen-allergic patients with or without pollen-related food allergy syndrome. (**a**) Prevalence in patients with PFAS; (**b**) prevalence in patients without PFAS; (**c**) median sIgE in patients with PFAS; (**d**) median sIgE levels in patients without PFAS. The sIgE assay was carried out by ImmunoCAP^TM^. Statistical analysis: Fisher’s exact and Mann–Whitney U tests; *p*-values *, 
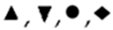
: <0.05; **: *p* = 0.05. Student’s *t*-test; 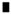: *p*-value < 0.05. Abbreviations: BPA, birch pollen-allergic patients; GPA, grass pollen-allergic patients; MPA, mixed pollen-allergic patients; MUXF3, CCD cross-reactive carbohydrate determinant; PFAS, patients with pollen-related food allergy syndrome; no PFAS, patients without pollen-related food allergy syndrome.

In GPA (Table 5), the co-sensitization to rBet v1 and two profilins [column A] significantly increased the prevalence of PFAS and OAS compared with that of GPA not sensitized to rBet v 1 and profilins [column E]. Respiratory disease severity was identical and rPhl p 1, nPhl p 4, rPhl p 5, and rPhl p 2 IgE levels were similar. GPA sensitized to rBet v 1 without co-sensitization to profilins [column B] had the same respiratory and food allergy phenotypes as GPA not sensitized to Bet v 1 [column E].

The phenotype and biological data for profilin(s) un- vs. mono- vs. polysensitized GPA patients are shown in Table 6. Polysensitized GPA tended to have a larger prevalence of PFAS (66.7%, *p* = 0.057), and a longer duration of PFAS and rhinitis; however, respiratory disease and PFAS severity was similar in the three groups. The prevalence of sensitization to rBet v 1 and rBet v 1-IgE levels were not significantly different. Profilin-polysensitized GPA did not have higher rPhl p 12- or rBet v 2 IgE levels compared with monosensitized GPA (*n* = 2, both sensitized to rBet v 2), but showed widely distributed values) (Table S5).

### 3.6. Correlation Between Levels with Profilins IgE (rBet v 2 and rPhl p 12) in and Between Grass Pollen-Allergic Patients (GPA) and Mixed Pollen-Allergic Patients (MPA)

In GPA, Spearman’s rho rank correlation between rPhl p 12 and rBet v 2 was positive, statistically significant, and very large (rho = 0.99, S = 74.33, *p* < 0.001). In MPA, there was a positive correlation (Spearman’s rho rank correletaion) between rPhl p 12 and rBet v2, which was statistically significant and very large (rho = 0.85, S = 918.62, *p* < 0.001). To compare the two correlations, after Fisher’s transformation (z1 = 2.6467 and z2 = 1.2562, SE 0.2582), statistical analysis yielded a Z value of = 5.39 (considerably above 1.96, corresponding to a 5% error risk), *p* < 0.0001. There is a good correlation between rPhl p 2- and rBet v 2 IgE in each group, but the correlation is stronger in GPA compared to MPA.

## 4. Discussion

Our study explored the IgE sensitization profile of patients allergic to birch, grass, or mixed pollens, and the relevance of the molecular sensitization (to allergen components) to respiratory and pollen-related food allergy phenotypes.

In the Belgian population of patients strictly allergic to *Betulaceae* or to *Poaceae* with AARC, approximatively 50% of patients allergic to one pollen family had a positive SPT to the other pollen family. In BPA, no sensitization to profilins was detected. A major role of the IgE sensitization to Bet v 1 was observed to play a major role in the severity of respiratory allergic disease and cross-reactive PFAS, particularly in BPA and MPA. The prevalence of PFAS among GPA also seemed to be driven by sensitization to Bet v 1, but with less severe symptoms. Co-sensitization to profilins and Bet v 1 increased the prevalence of PFAS in GPA, but only when compared to patients not sensitized to either type of allergen. Profilin-polysensitized GPA patients tended to have a higher prevalence of PFAS, together with a longer duration of PFAS and rhinitis compared with profilin mono- and un-sensitized GPA but with similar rBet v 1 sensitization and symptom severity. We studied populations with real clinical pollen allergy, not solely based upon pollen sensitization, in line with the rationale for outpatient clinic assessments.

Among patients living in Belgium suffering from pollen-induced AARC, GPA and MPA were the main groups, as seen in other countries. As observed in other studies, seasonal rhinitis was the most frequent respiratory symptom in the three groups of pollen-allergic patients [4,5,6,7,25]. Patient distribution in the three groups was consistent with that of European studies [1,2,5,6,26,27,28]. During our consecutive recruitment process over a limited period, a small number of strictly BP-allergic patients were included. This was due to the growing trend of overlapping pollen seasons associated with a high prevalence of co-sensitization and co-allergy to the other pollen studied.

Our BPA and GPA were rarely (10%) monosensitized to aeroallergens. Different factors influenced this result, including age (mainly adults), geographical distribution of patients, and the number of skin-tested allergens in particular [6,29]. In vitro, we observed that sensitization to the other studied pollen was independent of cross-reactivity to profilins. Similar results were observed by others in Belgium [30]. This justified the important distinction in the study between pollen sensitization or allergy.

Analysis of the prevalence of IgE sensitization to BP and TGP allergens showed that our Belgian GPA population seemed closer to Southern populations for minor allergens (rPhl p 7, rPhlp 12, rBet v 2, and rBet v 4) [3,12,19,31,32,33,34]. It was different for the major grass pollen allergens. The rPhl p 2 and rPhl p 6 components were major allergens resulting from geographical distribution and the level of exposure to the variety of the grass species [3,25,32,34]. The prevalence of sensitization to rPhl p 1 and nPhl p 4 was comparable to that of populations in Central and Northern Europe [33,35]. The coexistence of several major allergens in GPA was consistent with a clinical allergy to TGP in a predominantly adult population [36]. The prevalence of sensitization to rBet v 1 in our GPA (48%) corresponded to intermediate European BP exposure [1,2,25,32,33]. As seen in Northern populations, all of our BPA had significant levels of BP-IgE and rBet v 1-IgE [4,34,37]. None were sensitized to profilins, CCD and polcalcins, as observed in other reports [4,25,32]. Another Belgian study, published in 2004 and performed on 33 BPA (same diagnostic criteria), documented the absence of sensitization to Bet v 2 and CCD [32]. The same authors reported another study in 2010, performed on 37 BPA, and observed a low prevalence of sensitization to polcalcin (5–10%) but the mono-allergy diagnosis to BP was not clear [38]. The lack of sensitization to profilins in our BPA does not appear to be due to the size of the population. Moreover, the clinical relevance of polcalcin remains controversial [19].

Studies have established a correlation between aeroallergen _s_IgE level and the probability of clinical relevance [36,39]. In our study, and as reported in others, this was observed for rBet v 1 in BPA and MPA. This link was suggested for rPhl p 1, rPhl p 5b, nPhl p 4 and rPhl p 6 in GPA while the median level of rBet v 1-IgE remained low [3,11,25,31,34]. In MPA, rBet v 1 was the first major allergen with the highest median IgE level followed by rPhl p 1, nPhl p 4 and rPhl p 5b at levels nonetheless lower than in GPA [25].

The rhinitis was significantly more severe, and asthma more prevalent in BPA and MPA compared to GPA patients sensitized to rBet v 1 with no co-sensitization to profilins according to significantly higher levels of rBet v 1-IgE in BPA and MPA. This could result from the increased pollination of birch trees, length of pollination of cross-reactive *Betulaceae* and larger amounts of breathable fine particles containing the highly inflammatory Bet v 1 allergen [4,7,9,10,34].

In our study, 58% of the pollen-allergic population with respiratory symptoms had PFAS (80% of BAP, 76% of MAP and only 32% of GPA). Our PFAS population (largely represented by BPA and MPA) was also older, with later onset of respiratory disease compared to those without PFAS. These findings were not due to effect size (large and medium to large, respectively). These results were not observed in Southern Europe where plant food allergies were more often linked to profilin(s) or nsLTP sensitization [17]. Logistic regression analyses documented the increased and significant risk of developing PFAS among older and asthmatic patients with a pollen allergy (and vice versa). As reported by other authors, our study did not correlate this finding with the severity or a longer duration of respiratory symptoms [13,17,40]. That could result from the absence of association with other allergic respiratory diseases in our population. The OAS was the most frequent clinical symptom of PFAS in the three groups (>90%). PFAS patients also displayed higher IgE levels for BP and rBet v1 than patients without PFAS, in relation to the higher proportion of patients sensitized and allergic to BP and rBet v 1, corroborating previous reports [4,40]. Although the rBet v 1-IgE levels remained fairly low in GPA, the prevalence of sensitization to rBet v 1 was significantly higher in GPA with PFAS (with a significantly higher level of BP-IgE) compared to those without PFAS. Profilin IgE levels in GPA were lower compared to rBet v 1, and no other relevant allergens (e.g., LTP, TLP, storage proteins, isoflavone reductase) were identified using the ImmunoCAP^TM^ ISAC assay. No systemic reactions were reported, in contrast to Southern populations with more frequent reports of sensitization to nsLTP [17]. Food anaphyactic reactions due to Bet v 1-IgE sensitization in BPA have been progressively recorded since the beginning of the 21st century in our country [41]; the shortened recruitment period and the small size of our population were probably limiting factors. A few cases of angioedema and asthma symptoms have also been further observed in MPA and BPA patients ingesting or peeling cherries, carrots, and apples, or consuming soy milk, in line with previous reports [41,42,43]. The implicated foods (apple, other Rosaceae, kiwi) identified by our GPA with PFAS were not typically those reported by GPA with cross-reactive food allergy in relation to IgE sensitization to profilins, i.e., tomato, paprika, orange, melon, banana, and watermelon [15,17,44]. This later finding further reinforces the relevance of PR-10 proteins in GPA patients with PFAS.

Respiratory symptom severity in pollen-allergic patients was not influenced by the sensitization to profilins, in contrast to previous studies [15,20,21]. This was specifically documented for profilin-polysensitized GPA even though they tended to have a longer duration of rhinitis. However, GPA co-sensitized to rBet v 1 and at least two profilins had PFAS more often than those who were not co-sensitized, and symptoms were not more severe. There was no evidence linking the development of profilin sensitization to GP allergy severity. However, our co-sensitized rBet v 1-profilin GPA population was too small to confirm any relationship with higher rBet v 1-IgE values. The latter could have then also explained the increased prevalence of PFAS.

In the profilin-sensitized GAP, the number of patients was insufficient to perform statistical analysis. There was no argument to suspect a higher level of rBet v2-rPhl p 12-IgE in profilin-polysensitized patients. We noted that the profilin monosensitization in GAP was linked to the presence of rBet v 2 IgE in two patients highly sensitized to rBet v 1 too. It would be interesting to observe on larger GAP populations if the rBet v 2 monosensitization is observed only in patients with high rBet v 1 and/or BP IgE levels, and if Bet v 2, in the absence of cross-reactivity to other profilin(s), is clinically more relevant. We cannot rule out the possibility that this was due to chance. The correlation between the values of rBet v 2-IgE and rPhl p12-IgE in GPA was excellent and better than in MPA. The significant correlations reflect cross-reactivity between profilins, but the comparison of the two correlations revealed a significant difference suggesting a higher power of sensitization from Bet v 2 in GPA. The rBet v2-IgE level was higher in GPA than in MPA while the BP-IgE levels were largely higher in MPA than GPA.

The level of rPhl p 2 IgE was lower in pollen-allergic patients with PFAS including GPA, even though rPhl p2 was a major allergen in GPA. However, our population was insufficient (medium effect size) to document significant features associated with the combination low rPhl p 2-IgE and the presence of PFAS. All of these findings need to be confirmed by larger studies in the future.

Limitations of the study:

- The short inclusion period with reduced populations may have limited the identification of sensitization to minor allergens, particularly in BPA, together with the number of severe clinical reactions in patients with PFAS. The small sample size of the populations limited the power of statistical analyses (1-β below 0.8), reducing the ability to detect a real effect. The non-statistically significant differences observed in these populations could become significant in larger groups. For the statistically different results with medium-large to large effect sizes, the observed differences were scientifically significant.
- The clinical implications of the IgE sensitization to profilins may have been underestimated due to the limited populations and non-significant results.
- The study results offer a snapshot of the situation in 2010–2011 period. A contemporary study with the same design on larger populations could potentially lead to different results (particularly in terms of profilins sensitization, severity of symptoms, and identification of other major allergens). Our changing times and the increasing effects of climate change could be associated with modifications in sensitization profiles.

## 5. Conclusions

This study showed that the severity of rhinitis, together with the prevalence of asthma and respiratory symptoms associated with food ingestion/peeling all increased in birch- or mixed pollen-allergic patients sensitized to Bet v 1. Conversely, profilin sensitization, observed at low levels, was only associated with a higher prevalence of food allergy in GPA patients co-sensitized to Bet v1, but there was no correlation with symptom severity.

## Figures and Tables

**Figure 1:**
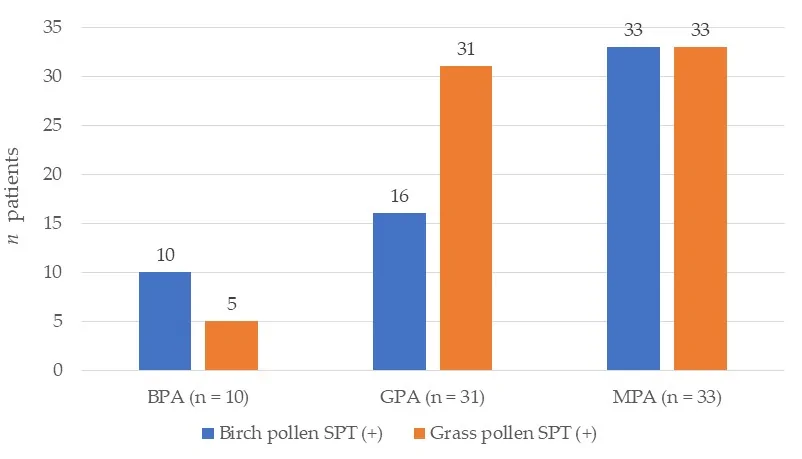
Prevalence of IgE sensitization to birch and grass pollen extracts, evaluated by skin-prick tests in the 3 groups of pollen-allergic patients (*n* = 74). Abbreviations: BPA, birch pollen-allergic patients; GPA, grass pollen-allergic patients; MPA, mixed pollen-allergic patients, SPTs, skin prick tests. Based on SPT, half of BPA are sensitized to grass pollen and half of GPA are sensitized to pollen of Betulaceae.

**Figure 2:**
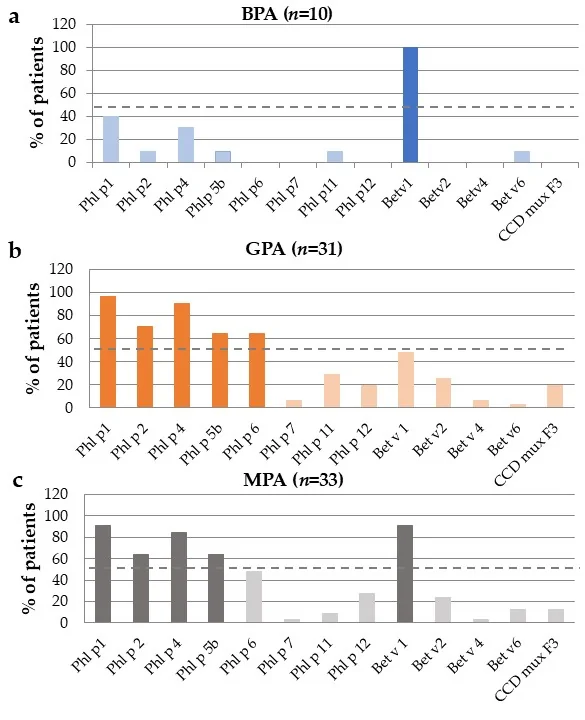
Prevalence of serum IgE sensitization to pollen allergen components in the 3 groups of pollen-allergic patients (*n* = 74): (**a**) birch pollen-allergic patients (BPA); (**b**) grass pollen-allergic patients (GPA); (**c**) mixed pollen-allergic patients (MPA). Abbreviation: CCD mux F3: cross-reactive carbohydrate determinant. The major allergens in each group appear darker. The dotted line represents 50% sensitization. The IgE assay was performed using ImmunoCAP^TM^.

**Figure 3:**
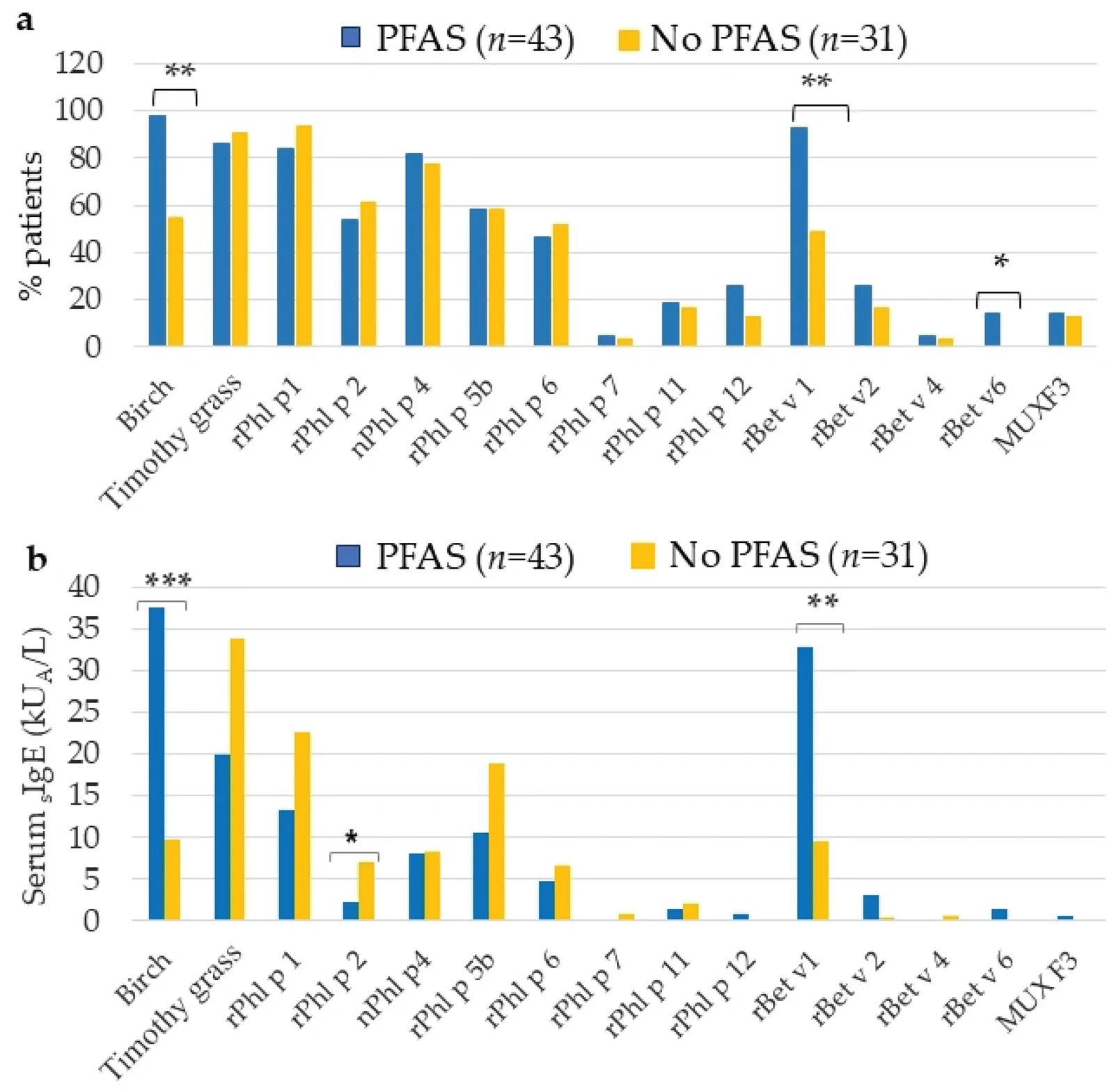
IgE sensitization to pollens and allergen components in pollen-allergic patients with or without pollen-related food allergy syndrome (*n* = 74). (**a**) prevalence; (**b**) mean levels of sIgE. The sIgE assay was performed by ImmunoCAP^TM^. Abbreviations: MUXF3, CCD cross-reactive carbohydrate determinant; PFAS, patients with pollen-related food allergy syndrome; No PFAS, patients without pollen-related food allergy syndrome. Statistical analysis: Student’s *t* test and Chi^2^; * *p* < 0.05; ** *p* < 0.0001; *** *p* < 0.00005.

**Table 1:** Demographic data and clinical characteristics of pollen-allergic populations with allergic rhinitis and/or conjunctivitis and/or asthma (AARC).

	BPA	GPA	MPA	All	*p*-Value
*n* (%) Gender M/F Age (yrs)	10 (13.5) 5/5 40.8 [35.5–53.4]	31 (41.9) 18/13 28.5 [17.1–36.7]	33 (44.6) 15/18 36 [23.6–41.4]	74 (100) 38/36 34 [19.5–40.9]	NS **0.01**
Rhino-(conjunctivitis) *n* (%) Gender M/F Age (yrs) Duration of disease (yrs) Severity (ARIA) *n* (%): Light intermittent Moderate-severe intermittent Mild persistent Moderate-severe persistent	9 (90) 4/5 42 [38.6–56.5] 15 [9.8–21.8] 1 0 0 8 (88.9)	31 (100) 18/13 28.5 [17.1–36.7] 9 [5–20] 3 6 2 20 (64.5)	33 (100) 15/18 36 [23.6–41.4] 10 [6–20] 5 3 1 24 (72.7)	73 (98.6) 37/36 34.1 [19.5–41.1] 10 [5–20] 9 9 3 52 (71.2)	NS NS **0.01** NS 0.08
Asthma *n* (%) Gender Age (yrs) Duration of disease (yrs) Severity (GINA) *n* (%): 1 2 3 4 unknown	9 (90) 4/5 42 [34.5–56.5] 8 [1–18] 3 (33.3) 4 (44.5) 1 (11.1) 0 1 (11.1)	13 (41.9) 7/6 25.6 [13.9–38.5] 5 [1–10] 9 (69.2) 2 (15.4) 2 (15.4) 0 0	21 (63.6) 10/11 36.9 [28–43] 8 [1–11] 13 (61.9) 7 (33.3) 0 1 (4.8) 0	43 (58.1) 21/22 36.5 [18.6–42.1] 6 [1–11] 25 (58.2) 13 (30.2) 3 (7) 1 (2.3) 1 (2.3)	**<0.05** NS 0.09 NS NS

Abbreviations: BPA, birch pollen-allergic patients; GPA, grass pollen-allergic patients; MPA, mixed (birch/grass) pollen-allergic patients; NS, not significant (*p*-value > 0.05). Results are expressed as median [25–75 interquartile range] values, or number and (%). Statistical analysis was performed using Kruskal–Wallis and Fisher’s exact tests. Significant *p*-values (*p* ≤ 0.05) are shown in bold font, while near-significant values are underlined.

**Table 2:** Demographic data and clinical characteristics of pollen-allergic patients (birch, grass, and mixed pollen) with allergic rhinitis, and/or conjunctivitis, and/or asthma, and plant-related food allergy.

	BPA	GPA	MPA	All	*p*-Value
**Plant Food allergy** *n* (%) Gender M/F Age (yrs) Duration of food allergy (yrs) Age at onset of respiratory disease (yrs) Food allergy symptoms *n* (%): OAS Other digestive Respiratory Contact urticaria	8 (80) 3/5 43 [39.3–58] 9 [3–16.5] 31.5 [21.7–34.8] 8 (100) 1 (12.5) 4 (50) 3 (37.5)	10 (32.3) 5/5 33.7 [27.1–40.3] 3 [1.7–5.5] 5.5 [14.4–17.8] 9 (90) 0 (0) 0 (0) 0 (0)	25 (75.8) 12/13 36.9 [28.7–41.5] 5 [1–10] 21.4 [12.5–28.6] 24 (96) 4 (16) 4 (16) 5 (20)	43 (58.1) 20/23 38.6 [29.1–43.5] 5 [1–10] 18.7 [12.5–30.5] 41 (95.4) 5 (11.6) 8 (18.6) 8 (18.6)	**<0.005** NS NS NS NS NS NS **<0.01** NS
**Plant Food allergy and asthma** *n* (%) Gender M/F Age (yrs) Duration of asthma (yrs) Severity of asthma (GINA) *n* (%): 1 2 3 4 unknown Rhinitis *n* (%) Severity (ARIA) *n* (%): Moderate-severe persist. Duration of rhinitis (yrs) Duration of food allergy (yrs) Food allergy symptoms *n* (%): OAS Other digestive Respiratory Contact urticaria	7 (70) 2/5 44 [40.3–59.5] 10 [4.5–19] 3 (42.8) 2 (28.6) 1 (14.3) 0 1 (14.3) 7 (100) 7 (100) 15 [10.5–27] 10.5 [0.9–0] 7 (100) 1 (14.3) 4 (66.7) 3 (42.9)	4 (12.9) 3/1 36.2 [33.3–37.8] 4.5 [3.1–9.3] 2 (50) 2 (50) 0 0 0 4 (100) 3 (75) 20 [15.8–25.3] 3 [1.8–4] 4 (100) 0 0 0	19 (57.6) 10/9 39.4 [29.1–43.6] 8 [1–10.5] 11 (57.9) 7 (36.8) 0 1 (5.3) 0 19 (100) 14 (73.7) 12 [5.5–24.5] 5 [1–10] 18 (94.7) 3 (15.8) 3 (15.8) 5 (26.3)	30 (40.5) 15/15 39.7 [30.5–44.1] 8 [1–11,] 16 (53.3) 11 (36.7) 1 (3.3) 1 (3.3) 1 (3.3) 30 (100) 24 (80) 14 [6–28] 4.5 [0.9–10] 30 (100) 4 (13.3) 7 (23.3) 8 (26.7)	NA NS NS NS NS NA NS NS NS NS NS **<0.05** NS

Abbreviations: BPA, birch pollen-allergic population; GPA, grass pollen-allergic population; MPA, mixed (birch/grass) pollen-allergic population; NA, not applicable; NS, not significant (*p*-value > 0.05); OAS, oral allergy syndrome. The upper part of the table shows data of patients with plant food allergy in the 3 pollen-allergic groups; the lower part presents the data for patients in the 3 groups with asthma and plant food allergy. Data are expressed as median [25–75 interquartile range] values or number (%). Statistical tests used: Kruskal–Wallis and Fisher’s exact tests. Significant *p*-values (*p* ≤ 0.05) are shown in bold font.

**Table 3:** _s_IgE levels and prevalence of serum IgE sensitization to pollens and allergen components (ImmunoCAP^TM^ assay) in the 3 groups of pollen-allergic patients.

	BPA (*n* = 10)		GPA (*n* = 31)		MPA (*n* = 33)	
	IgE Level kU_A_/L	% IgE (*n*)	IgE Level kU_A_/L	% IgE (*n*)	IgE Level kU_A_/L	% IgE (*n*)
Total IgE	151.5 [55–357]		257 [131–355]		131 [75–268]	
Birch	36 [13.6–48.9]	100 (10)	1.2 [0.1–6.9]	54.8 (17)	25.2 [12.3–46.2]	97 (32)
Timothy grass	0.14 [<0.1–1]	30 (3)	27.5 [4.9–77.7]	100 (31)	9.5 [2.6–17.5]	93.9 (31)
rPhl p 1	<0.1 [0.1–0.5]	40 (4)	15.7 [3.7–43.4]	**96.8** (30)	4.8 [1.4–11.4]	**90.9** (30)
rPhl p 2	<0.1 [<0.1–<0.1]	10 (1)	1.88 [0.2–8.3]	**71** (22)	0.71 [<0.1–3.6]	**57.6** (19)
nPhl p 4	0.12 [<0.1–0.3]	30 (3)	7.99 [1.6–18]	**90.3** (28)	2.97 [1–5.7]	**84.8** (28)
rPhl p 5b	<0.1 [<0.1–<0.1]	10 (1)	13.1 [<0.1–40.1]	**67.7** (21)	2.13 [<0.1–7.8]	**63.6** (21)
rPhl p 6	<0.1 [<0.1–<0.1]	0	4.01 [<0.1–14.5]	**64.5** (20)	0.3 [<0.1–2.7]	48.5 (16)
rPhl p 7	<0.1 [<0.1–<0.1]	0	<0.1 [<0.1–<0.1]	6.5 (2)	<0.1 [<0.1–<0.1]	3 (1)
rPhl p 11	<0.1 [<0.1–<0.1]	10 (1)	<0.1 [<0.1–0.7]	29 (9)	<0.1 [<0.1–<0.1]	9.1 (3)
rPhl p 12	<0.1 [<0.1–<0.1]	0	<0.1 [<0.1–0.2]	19.4 (6)	<0.1 [<0.1–0.4]	27.3 (9)
rBet v 1	36.2 [12.6–62.2]	**100** (0)	0.28 [<0.1–7.5]	48.4 (15)	22.2 [11.3–40]	**90.9** (30)
rBet v 2	<0.1 [<0.1–<0.1]	0	<0.1 [<0.1–0.3]	25.8 (8)	<0.1 [<0.1–0.3]	24.2 (8)
rBet v 4	<0.1 [<0.1–<0.1]	0	<0.1 [<0.1–<0.1]	6.5 (2)	<0.1 [<0.1–<0.1]	3 (1)
rBet v 6	<0.1 [<0.1–<0.1]	10 (1)	<0.1 [<0.1–<0.1]	3.2 (1)	<0.1 [<0.1–<0.1]	12.1 (4)
CCD-MUXF3	<0.1 [<0.1–<0.1]	0	<0.1 [<0.1–<0.1]	19.4 (6)	<0.1 [<0.1–13.4]	12.1 (4)

Abbreviations: BPA, birch pollen-allergic patients; CCD, cross-reactive carbohydrate determinant; GPA, grass pollen-allergic patients; MPA, mixed (birch/grass) pollen-allergic patients. The prevalence of _s_IgE sensitizations is expressed as % (number) and the results for _s_IgE level are expressed as median [25–75 interquartile range] values in kU_A_/L. The major allergens are shown in bold font (% of positive sIgE > 50%).

**Table 4:** Respiratory phenotype and key results of biological tests of pollen-allergic patients with and without plant-related food allergy syndrome (PFAS).

	Patients with PFAS	Patients Without PFAS	All	*p*-Value
*n* (%) Age at inclusion time Gender M/F Age at onset of respiratory allergy Group of patients with pollen allergy: Birch (BPA) Grass (GPA) Mixed (MPA)	43 (58.1) 36.4 ± 12.4 20/23 21.3 ± 11.8 8 (80%) 10 (32%) 25 (76%)	31 (41.9) 24.6 ± 13.6 18/13 14.2 ± 7.6 2 (20%) 21 (68%) 8 (24%)	74 (100) 31.5 ±14.1 38/36 18.4 ± 10.6 10 31 33	**<0.0005** NS **<0.005** **<0.001**
Rhinitis *n* (%) Severity of rhinitis ARIA: Light intermittent Moderate-severe intermittent Light persistent Moderate-severe persistent Duration of rhinitis (yrs)	43 (100) 7 3 2 31 13.3 ± 10.7	30 (96.8) 2 6 1 21 10.2 ± 10.2	73 (98.7) 9 9 3 52 12 ± 10.5	NS NS NS
Asthma *n* (%) Severity of asthma (GINA): 1 2 3 4 Unknown Duration of asthma (yrs)	30 (69.8) 16 (53.3) 11 (36.7) 1 (3.3) 1 (3.3) 1 (3.3) 9.2 ± 9.1	13 (41.9) 9 (69.2) 2 (15.4) 2 (15.4) 0 0 6.4 ± 6.4	43 (58.1) 25 (58.1) 13 (30.2) 3 (7) 1 (2.3) 1 (2.3) 8.4 ± 8.4	**<0.05** NS NS
Biological data: Tot IgE Birch IgE rBet v 1 IgE rBet v 2 IgE rBet v 6 IgE rPhl p 12 IgE rPhl p 2 IgE	347.7 ± 771.4 37.5 ± 34.1 32.8 ± 30.5 3.1 ± 15.1 1.4 ±.9 0.8 ± 1.9 2.2 ± 3.4	284.8 ± 207 9.7 ± 18.2 9.6 ± 16 0.3 ± 0.6 0.1 ± 0.04 0.3 ± 0.5 7.0 ± 11.8	321.3 ± 600.8 25.9 ± 31.6 23.1 ± 27.8 1.9 ± 11.5 0.9 ± 4.5 0.6 ±.5 4.2 ± 8.3	NS **<0.00005** **<0.0001** NS NS NS **<0.05**

Abbreviation: NS, non-significant (*p*-value > 0.05). Age and duration are expressed in years and shown as mean ± SD. The _s_IgE levels results are expressed in kU_A_/L (ImmunoCAP^TM^ assay) and shown as mean ± SD. Statistical analysis for parametric tests: Student’s t and Chi^2^ tests. Differences statistically significant are shown in bold font.

**Table 5:** Respiratory and food allergy phenotypes of grass pollen-allergic patients according to the combination of IgE sensitization to rBet v 1 and profilin(s) (rBet v 2, rPhl p 12).

GPA (*n* = 31)	Bet v 1, Bet v 2, Phl p 12 (+) [A]	Bet v 1 (+), Phl p 12 and Bet v 2 (−) [B]	Bet v 1 and Bet v 2 (+), Phl p 12 (−) [C]	Bet v 1 (−), Bet v 2 and Phl p 12 (+) [D]	Bet v1, Bet v 2 and Phl p 12 (−) [E]	Bet v 1, Phl p 12 (−) and Bet v 2 (+) [F]	*p*-Value [A], [B], [D], [E]
*n* (%)	4 (12.9)	10 (32.3)	1 (3.2)	2 (6.5)	13 (41.9)	1 (3.2)	
Rhinitis *n* (%) Severity ARIA: Light intermittent Moderate-severe intermittent Light persistent Moderate-severe persistent Duration of rhinitis (yrs)	4 (100) 0 0 0 4 (100) 13.5 [6.8–23.8]	10 (100) 3 (30) 1 (10) 1 (10) 5 (50) 7 [5–19]	1 (100) 0 1 0 0 0.6 [0.6–0.6]	2 (100) 0 0 0 2 (100) 17.5 [16.3–19]	13 (100) 0 4 (30.8) 1 (7.7) 8 (61.5) 10 [6–18]	1 (100) 0 0 0 1 4 [4–4]	NS NS
Asthma *n* (%) Severity GINA: 1 2 3 Duration of asthma (yrs)	2 (50) 1 (50) 0 1 (50) 12.5 [8.6–16.6]	3 (30) 2 (66.7) 1 (33.3) 0 4 [2.2–13]	0	1 (50) 1 (100) 0 0 8 [7–9]	7 (53.6) 5 (71.4) 1 (14.3) 1 (14.3) 1 [0.8–9.5]	0	NS NS
PFAS *n* (%) Duration of PFAS (yrs)	3 (75) ^a^ 7 [6- 7.5]	4 (40) 1.8 [1.2-2.4]	1 (100) 0	1 (50) 3	1 (7.7) ^a^ 0.6	0 0	**<0.05** NS
PFAS symptom OAS Other digestive Respiratory Urticaria	3 ^b^ 0 0 0	4 1 0 1	1 0 0 0	0 1 0 0	1 ^b^ 0 0 0	0 0 0 0	**<0.05** NS NA NS
IgE (kUA/L) _Tot_ IgE rBet v 1 rBet v 2 rPhl p 12 BP TGP rPhl p 1 nPhl p 4 rPhl p 5b rPhl p 2	136 [110–196] 17.2 [9–29.4] ^e^ 1.35 [0.9–3.5] ^d,e^ 1.6 [1.2–2.8] ^d,e^ 21.9 [7.6–38.4] ^e^ 41.4 [22.6–67.4] 16.7 [6.8–44.9] 10.63 [7.7–34.2] 18.1 [7.6–45.3] 1.59 [1.2–2.63]	232.5 [151–349] 5.6 [1.9–24] ^c^ 0.1 [0.1–0.1] ^d,f^ 0.1 [0.1–0.1] ^d,g^ 4.54 [1.8–9.1] 22.6 [3.8–86.8] 11 [1.3–50.5] 4.52 [1.15–11.7] 10 [0.1–37.2] 2.42 [0.39–3.94]	417 [417–417] 100 [100–100] 99 [99–99] 0.3 [0.3–0.3] 100 [100–100] 43 [43–43] 37 [37–37] 25.5 [25.5–25.5] 0.1 12.3 [12.3–12.3]	384.5 [315–454] 0.1 [0.1–0.1] 2.5 [2.1–2.9] 2.1 [1.8–2.4] 2.2 [1.7–2.7] 42 [34.4–49.7] 15.7 [15.1–16.2] 10.8 [9.4–12.1] 20 [19.1–21] 6 [3.03–8.9]	289 [125–349] 0.1 [0.1–0.1] ^c,e^ 0.1 [0.1–0.1] ^e^ 0.1 [0.1–0.1] ^e^ 0.1 [0.1–0.1] ^e^ 20.8 [4–77.1] 13.3 [3.4–5.9] 4.9 [1.3–16.4] 2.6 [0.1–37.1] 1.8 [0.1–7.2]	652 0.2 0.4 ^f^ 0.3 ^g^ 4.4 100 100 27.2 85 47.1	NS **<0.0001** **<0.0001** **<0.0001** **<0.0001** NS NS NS NS NS

Abbreviations: BP, birch pollen; GPA, grass pollen-allergic population; NA: not applicable; NS, not significant; OAS, oral allergy syndrome; PFAS, pollen-related food allergy syndrome; TGP, Timothy grass pollen. The results for the duration of progression were expressed by median [25–75 interquartile range] values. Positivity was defined by component allergic IgE level > 0.35 kUA/L (ImmunoCAP^TM^ assay). Statistical analysis was performed using Fisher’s exact test and the Kruskall-Wallis or Mann–Whitney U tests. Significant *p*-values (*p* ≤ 0.05) are shown in bold font. Statistically significant differences were observed for the group-by-group analyses, *p*-values: ^a^, ^b^: *p* < 0.005; ^c^: *p* < 0.0001; ^d^: *p* < 0.001; ^e^: *p* < 0.0005; ^f^, ^g^: *p* < 0.01. A to F are used to identified the columns of data.

**Table 6:** Comparison of the clinical and biological phenotypes of profilin(s) un-, mono- and poly-sensitized grass pollen-allergic patients (GPA).

GPA *n* = 31	Profilin Polysensitized *n* = 6	Profilin Monosensitized *n* = 2	Profilin Un-Sensitized *n* = 23	*p*-Value
Age (yrs) Gender M/F	35.1 [24–39.6] 4/2	38.3 [31.9–44.6] 0/2	25.6 [14.5–35.5] 14/9	NS NS
Rhinitis: *n* (%) Severity ARIA: Light intermittent Moderate-severe intermittent Light persistent Moderate-severe persistent Duration of rhinitis (yrs):	6 (100) 0 0 0 6 17.5 [9–20]	2 (100) 0 1 0 1 2.3 [1.5–3.2]	23 (100) 3 5 2 13 9 [5–19]	NA NS 0.06
Asthma *n* (%) Severity GINA: Level I Level II Level III Duration of asthma (yrs):	3 (50) 2 0 1 6 [5.5–13]	0 NA	10 (43.5) 7 2 1 2.5 [0.6–9.8]	NS NS NS
PFAS Symptoms *n* (%): OAS Other digestive Respiratory Contact urticaria Duration of PFAS (yrs):	4 (66.7) 3 1 0 0 7 [6–7.5]	1 (50) 1 0 0 0 0.6 [0.6–0.6]	5 (21.7) 5 1 0 1 2.5 [1.7–3]	0.057 NS NS NA 1 0.061
Biological data: _tot_IgE BP IgE TGP IgE rPhl p 12 IgE rBet v 2 IgE rBet v 1 IgE rPhl p 1 IgE nPhl p 4 IgE rPhl p 5b IgE rPhl p 2 IgE rBet v 1 IgE > 0.35 *n* (%)	195 [131–320.8] 7.6 [4.3–28.9] 4.6 [26.3–57.1] 1.6 [1.4–2.5] 1.6 [1.2–2.9] 8.6 [2.2–21.5] 15.7 [8.8–24.1] 10.7 [8.3–13.2] 20 [11.3–25.8] 1.59 [0.6–4.6] 4 (66.6)	535 [476–593] 52.2 [28.3–76.1] 71.5 [57.3–85.8] 0.27 [0.26–0.28] 49.6 [25–74.2] 50.1 [25.2–75.1] 68.4 [52.6–84.2] 26.4 [25.9–26.8] 42.4 [21.2–63.5] 29.7 [21–38.4] 1 (50)	257 [130.5–350.5] [0.1–2.9] 20.8 [3.6–77.7] 0.1 [0.1–0.1] 0.1 [0.1–0.1] 0.1 [0.1–3.88] 13.3 [3–43.4] 4.9 [1.4–14.7] 6.9 [0.1–40.1] 1.88 [0.1–5–6] 10 (43.4)	NS **0.01** NS **<0.00001** **<0.00001** NS NS NS NS NS NS

Abbreviations: BP, birch pollen; OAS, oral allergy syndrome; PFAS, pollen-related food allergy syndrome; TGP, Timothy grass pollen; NA, not applicable; NS, not significant. The profilin sensitization was defined by a level of rBet v 2 and/or rPhl p 12 IgE > 0.35 kU_A_/L in ImmunoCAP^TM^ assay. _Tot_IgE and _s_IgE levels are expressed by median [25–75 interquartile range] values in kU_A_/L. Statistical analysis with non-parametric tests (Kruskal–Wallis and Fisher exact tests). Bolding used for significant *p*-value (*p* ≤ 0.05) and underlining to emphasize quasi-significant *p*-value.

## Data Availability

Data are available on request from the corresponding author due to the language used for the construction of the database.

## Supplementary Materials

The following supporting information can be downloaded at: https://www.mdpi.com/article/10.3390/cells15201907/s1, Table S1. Statistical analysis performed on the sIgE levels to pollen allergens and their allergen components (ImmunoCAPTM assay) between the 3 groups of pollen-allergic patients. Abbreviations: BPA, birch pollen-allergic patients; CCD, cross-reactive carbohydrate determinant; GPA, grass pollen-allergic patients; MPA, mixed (birch/grass) pollen-allergic patients; NS, not significant. Statistical tests used: Kruskall-Wallis and Mann-Whitney U tests. Significant *p*-values (*p* ≤ 0.05) are shown in bold font. Near-significant *p*-value are underlined. The median values of sIgE levels are expressed in Table 3. Table S2. Manifestations of plant food allergy spontaneously reported by the patients according to the nature of the pollen allergy and food. Abbreviations: AARC: Allergic asthma and/or rhino-conjunctivitis; BPA, birch pollen-allergic patients; D, other digestive symptoms; GPA, grass pollen-allergic patients; MPA, birch and grass pollen-allergic patients; OAS, oral allergy syndrome; R, respiratory symptoms; U, contact urticaria. Exposure to raw food occured via the oral, respiratory or percutaneous route. Results are expressed in % for each group of pollen-allergic patients. Nuts include walnuts and hazelnuts. The other Rosaceae include pear, plum, apricot. The other digestive symptoms are oropharyngeal edema, epigastralgia, diarrhea, nausea and vomiting or abdominal cramp. The respiratory symptoms are cough, dyspnea, wheezing, chest tightness or laryngeal edema. Table S3. Prevalence and level of sIgE to allergen components for Thaumatin-Like, Cupin and Prolamin superfamilies (ImmunoCAP^®^ ISAC 103) in pollen-allergic patients with or without plant-related food allergy syndrome. Abbreviations: NS, not significant; PFAS, plant-related food allergy syndrome. The statistical analysis is performed with parametric tests: Chi^2^ and Student′s *t* tests. The IgE sensitization is defined by a level of sIgE > 0.3 ISU. Table S4. Influence of the IgE sensitization to rBet v 1 and at least 1 profilin (rBet v 2 and/or rPhl p 12) on the phenotype of the 3 groups of pollen-allergic patients. Abbreviation: BPA, birch pollen-allergic population; GPA, grass pollen-allergic population; MPA, mixed (birch/grass) pollen-allergic population; OAS, oral allergy syndrome, PFAS, Pollen-related food allergy syndrome. Positive sensitization was defined by a level of sIgE > 0.35 kUA/L (ImmunoCAPTM assay). Age and sIgE results are expressed by median [25–75 interquartile range] values. Significant *p*-value (*p* ≤ 0.05) are presented in bold font. Statistical analysis was performed with Fisher’s exact and Kruskal-Wallis or Mann-Whitney U tests.

## Abbreviations

The following abbreviations are used in this manuscript:

AARC	allergic rhinitis and/or conjunctivitis with/without asthma
BP	birch pollen
BPA	birch pollen-allergic patient(s)
CCD or CCD mux F3	cross-reactive carbohydrate determinant
GP	grass pollen
GPA	grass pollen-allergic patient(s)
MPA	mixed pollen-allergic patient(s)
OAS	oral allergy syndrome
PFAS	pollen-related food allergy syndrome
PFASs	pollen-related food allergy symptoms
Phl p	Phleum pratense
SPT	skin-prick test
SPPT	skin prick-prick test
TGP	Timothy grass pollen
