# Supplementary material for: Bet v 1 Allergen Is the Main Driver of Disease in Patients with Pollen Allergy in Belgium
Source: Cells. 2026 Oct 20;15(20):1907. doi: 10.3390/cells15201907 (PMC13703822; doi:10.3390/cells15201907)
Supplement: Supplementary file 1 [file cells-15-01907-s001.zip › Table S4.pdf]

Table S4. Influence of the IgE sensitization to rBet v 1 and at least 1 profilin (rBet v 2 and/or rPhl p 12) on the phenotype of the 3 groups of pollen-allergic patients. Abbreviation: BPA, birch pollen-allergic population; GPA, grass pollen-allergic population; MPA, mixed (birch/grass) pollen-allergic population; OAS, oral allergy syndrome, PFAS, Pollen-related food allergy syndrome. Positive sensitization was defined by a level of sIgE >0.35 kUA/L (ImmunoCAPTM assay). Age and sIgE results are expressed by median [25-75 interquartile range] values. Significant p-value (p≤0.05) are presented in bold font. Statistical analysis was performed with Fisher's exact and Kruskal-Wallis or Mann-Whitney U tests.

n=74	rBet v 1 (+), rBet v 2 and rPhl p 12 (-) *n* = 43				rBet v 1 (+), ≥1 profilin(s) (+) *n* = 13			rBet v 1 (-), ≥1 profilin(s) (+) *n* = 5			rBet v 1 and 2 profilins (-) *n* = 13
	BPA	GPA	MPA	*p*	GPA	MPA	*p*	GPA	MPA	*p*	GPA
n (%)	10 (23.3)	10 (23.3)	23 (53.4)		5 (38.5)	8 (61.5)		3 (60)	2 (40)		13
Median age	40.8 [35.5-53.4]	28.6 [22.5-31.3]	36.9 [28.8-41.5]	NS	39.9 [21.5-41.5]	28.5 [22-39.4]	NS	31.6 [28.6-35.1]	18.9 [13.9-23.8]	NS	17.6 [13.8-35.8]
**Rhinitis** *n* (%)	9 (90)	10 (100)	23 (100)		5 (100)	8 (100)		3 (100)	2 (100)		13 (100)
Severity (Aria): *n* (%)											
Light intermittent	1 (11)	3 (30)	3 (13)	NS	0	2 (25)		0	0		0
Moderate-severe intermittent	0	1 (10)	2 (9)	**<0.05**	1 (20)	1 (13)	NS	0	0	NS	4 (31)
Light persistent	0	1 (10)	1 (4)		0	0		0	0		1 (8)
Moderate-severe persistent	8 (89)	5 (50)	17 (74)		4 (80)	5 (63)		3	2		8 (62)
**Asthma** *n* (%)	9 (90)	3 (30)	15(65)		2 (40)	5 (63)		1 (33.3)	1 (50)		7 (54)
Severity (GINA):				**<0.05**			NS			NS	
1	3 (33)	2 (67)	7 (47)		1 (50)	5 (100)		1	1		5 (72)
2	4 (45)	1 (33)	7 (47)	NS	0	0	NS	0	0	NA	1 (14)
3	1 (11)	0	0		1 (50)	0		0	0		1 (14)
4	0	0	1 (7)		0	0		0	0		0
Unknown	1 (11)										
**PFAS** *n* (%)	8 (80)	4(40)	17(74)	NS	4 (80)	7 (87.5)	NS	1 (33.3)	1 (50)	NS	1 (7.7)
Symptoms:											
OAS	8(100)	4(100)	16(94)	NS	4 (100)	7 (100)	NS	0	1 (100)	NS	1 (100)
Other digestive	1 (13)	1 (25)	4 (24)	NS	0	1 (14)	NS	1 (100)	0	NS	0
Respiratory	4 (50)	0	4 (24)	**<0.05**	0	0	NS	0	0	NA	0

Contact urticaria	3 (38)	1 (25)	4 (24)	NS	0	1 (14)	0	0	NA	0
