# Supplementary material for: Bet v 1 Allergen Is the Main Driver of Disease in Patients with Pollen Allergy in Belgium
Source: Cells. 2026 Oct 20;15(20):1907. doi: 10.3390/cells15201907 (PMC13703822; doi:10.3390/cells15201907)
Supplement: Supplementary file 1 [file cells-15-01907-s001.zip › Table S1.pdf]

Table S1. Statistical analysis performed on the sIgE levels to pollen allergens and their allergen components (ImmunoCAPTM assay) between the 3 groups of pollen-allergic patients. Abbreviations: BPA, birch pollen-allergic patients; CCD, cross-reactive carbohydrate determinant; GPA, grass pollen-allergic patients; MPA, mixed (birch/grass) pollen-allergic patients; NS, not significant. Statistical tests used: Kruskall-Wallis and Mann-Whitney U tests. Significant p-values (p≤0.05) are shown in bold font. Near-significant p-value are underlined. The median values of sIgE levels are expressed in Table 3.

	**Comparison of the median sIgE levels**			
	**3 Groups *p*-value**	**Group by group *p*-value**		
		**BPA- GPA**	**BPA- MPA**	**GPA-MPA**
Total IgE	NS	NS	NS	0.054
Birch	**<0.0001**	**<0.0005**	NS	**<0.0001**
Timothy grass	**<0.0001**	**<0.0001**	**<0.0001**	**<0.05**
rPhl p 1	**<0.0001**	**<0.0001**	**<0.0001**	**<0.01**
rPhl p 2	**<0.001**	**<0.0005**	**<0.005**	NS
nPhl p 4	**<0.0001**	**<0.0001**	**<0.0001**	**<0.05**
rPhl p 5b	**<0.005**	**<0.005**	**<0.01**	NS
rPhl p 6	**<0.005**	**<0.005**	**<0.05**	**0.05**
rPhl p 7	NS	NS	NS	NS
rPhl p 11	NS	NS	NS	0.055
rPhl p 12	NS	NS	NS	NS
rBet v 1	**<0.0001**	**<0.0005**	NS	**<0.0001**
rBet v 2	NS	NS	**<0.05**	NS
rBet v 4	NS	NS	NS	NS
rBet v 6	NS	NS	NS	NS
CCD-MUXF3	NS	NS	NS	NS
