# Supplementary material for: Bet v 1 Allergen Is the Main Driver of Disease in Patients with Pollen Allergy in Belgium
Source: Cells. 2026 Oct 20;15(20):1907. doi: 10.3390/cells15201907 (PMC13703822; doi:10.3390/cells15201907)
Supplement: Supplementary file 1 [file cells-15-01907-s001.zip › Table S2.pdf]

Table S2. Manifestations of plant food allergy spontaneously reported by the patients according to the nature of the pollen allergy and food. Abbreviations: AARC : Allergic asthma and/or rhino-conjunctivitis ; BPA, birch pollen-allergic patients ; D, other digestive symptoms ; GPA, grass pollen-allergic patients ; MPA, birch and grass pollen-allergic patients ; OAS, oral allergy syndrome ; R, respiratory symptoms ; U, contact urticaria. Exposure to raw food occured via the oral, respiratory or percutaneous route. Results are expressed in % for each group of pollen-allergic patients. Nuts include walnuts and hazelnuts. The other Rosaceae include pear, plum, apricot. The other digestive symptoms are oropharyngeal edema, epigastralgia, diarrhea, nausea and vomiting or abdominal cramp. The respiratory symptoms are cough, dyspnea, wheezing, chest tightness or laryngeal edema.

**Pollen AARC with food allergy**	**BPA (*n*=8/10) 80 %**				**GPA (*n*=10/31) 32 %**				**MPA (*n*=25/33) 76 %**			
**Food allergic symptoms**	**OAS** %	**D** %	**R** %	**U** %	**OAS** %	**D** %	**R** %	**U** %	**OAS** %	**D** %	**R** %	**U** %
Apple	70		10	20	9.7				51.5	6	3	
Nuts	60				19.4				36.4	3		
Peach	20								48.5	9.1		
Cherry	40		10		3.2				42.4	6		
Strawberry	40			10								
Other Rosaceae	30		10	20	9.7							
Peanut					3.2				6		3	
Almond					3.2				9.1			
Banana	10				3.2				9.1			
Kiwi	20		10	10	9.7				27.3	3		
Carrot	10		10		3.2				24.2	6	3	3
Potato			10	10				3.2			3	12,1
Tomato					3.2			3.2	3			
Orange					6.4							
Melon					6.4	3.2			15.2			
Avocado					3.2							
Soy									3	6	3	
Water-melon									6			
Fig									3			
Pineapple									3			
Papaya									3			
Grapes									3	3		
Chicory									3			
