# Supplementary material for: Bet v 1 Allergen Is the Main Driver of Disease in Patients with Pollen Allergy in Belgium
Source: Cells. 2026 Oct 20;15(20):1907. doi: 10.3390/cells15201907 (PMC13703822; doi:10.3390/cells15201907)
Supplement: Supplementary file 1 [file cells-15-01907-s001.zip › Table S3.pdf]

Table S3. Prevalence and level of sIgE to allergen components for Thaumatin-Like, Cupin and Prolamin superfamilies (ImmunoCAP® ISAC 103) in pollen-allergic patients with or without plant-related food allergy syndrome. Abbreviations: NS, not significant; PFAS, plant-related food allergy syndrome. The statistical analysis is performed with parametric tests: Chi² and Student's t tests. The IgE sensitization is defined by a level of sIgE>0.3 ISU.

	Pollen allergic patients with PFAS (*n*=43)	Pollen allergic patients without PFAS (*n*=31)	p value	Pollen allergic patients with PFAS (*n*=43)	Pollen allergic patients without PFAS (*n*=31)	*p*-value
	Prevalence of IgE sensitization (%)			Level of sIgE (ISU): median (25-75 interquartile range) [min - max]		
nsLTP (Prolamin superfamily))						
nPru p 3	4,7	0	NS	0 (0 - 0) [0 - 2,4]	0 (0 - 0) [0 - 0]	NS
rCor a 8	2,3	0	NS	0 (0 - 0) [0 - 20]	0 (0 - 0) [0 - 0]	NS
rPar j 2	0	0	NS	0 (0 - 0) [0 - 0]	0 (0 - 0) [0 - 0]	NS
nArt v 3	2,3	0	NS	0 (0 - 0) [0 - 0,6]	0 (0 - 0) [0 - 0]	NS
2S Albumin (Prolamin superfamily)						
nAra h 2	0	0	NS	0 (0 - 0) [0 - 0]	0 (0 - 0) [0 - 0]	NS
rBer e 1	4,7	0	NS	0 (0 - 0) [0 - 17]	0 (0 - 0) [0 - 0]	NS
nSes i 1	0	0	NS	0 (0 - 0) [0 - 0]	0 (0 - 0) [0 - 0]	NS
Thaumatin-like protein						
nAct d 2	7	9,7	NS	0 (0 - 0) [0 -3,1]	0 (0 - 0) [0 - 3,9]	NS
7S Globulin (Cupin superfamily)						
nAra h 1	0	0	NS	0 (0 - 0) [0 - 0]	0 (0 - 0) [0 - 0]	NS
nGly m 5	0	0	NS	0 (0 - 0) [0 - 0]	0 (0 - 0) [0 - 0]	NS
11S Globulin (Cupin superfamily)						
nAra h3	2,3	0	NS	0 (0 - 0) [0 - 13]	0 (0 - 0) [0 - 0]	NS
nGly m 6	0	0	NS	0 (0 - 0) [0 - 0]	0 (0 - 0) [0 - 0]	NS
nCor a 9	2,3	0	NS	0 (0 - 0) [0 - 1,2]	0 (0 - 0) [0 - 0]	NS
rAna o 2	0	0	NS	0 (0 - 0) [0 - 0]	0 (0 - 0) [0 - 0]	NS
